# Proceedings of the Hahnemann Club of Toronto

**Published:** 1888-08

**Authors:** J. D. Tyrrell

**Affiliations:** Secretary-Treasurer


### PROCEEDINGS OF THE HAHNEMANN CLUB OF TORONTO.

Regular meeting of the Hahnemann Club was held on Wednesday evening, June 6th.

The sight of the vacant chair formerly occupied by our honored President, Dr. Hall, caused a feeling of gloom to pervade our meeting. We missed his genial face and valuable advice and criticism. It was moved by Dr. Tyrrell, seconded by Dr. Emory, that Dr. E. T. Adams be elected President for balance of club year; the motion was carried, and the Doctor, in flowing terms and faultless diction, gracefully acknowledged the honor conferred upon him.

The subject of convulsions will be continued at our next meeting.

The papers on *Convulsions* were read, as follow:

J. D. Tyrrell,
*Secretary-Treasurer.*

